# Clinical Characteristics, Complications, and Post-stent Survival in Patients Undergoing Palliative Oesophageal Stenting: A Retrospective Study

**DOI:** 10.7759/cureus.107613

**Published:** 2026-04-23

**Authors:** Nilanga Nishad, Sarala Janarthanan, Andreas Hadjinicolaou, Kumarini Basnayake, Joshua Elias, Vasitha Abeysuriya, Visula Abeysuriya, Januli Gamage, Ines Modollel, Dunecan Massey, Gareth Corbett

**Affiliations:** 1 Gastroenterology, Cambridge University Hospital NHS Foundation Trust, Cambridge, GBR; 2 Gastroenterology, Sheffield Teaching Hospitals NHS Foundation Trust, Sheffield, GBR; 3 Surgery, Faculty of Medicine, University of Kelaniya, Colombo, LKA; 4 Department of Immunology, Institute of Biochemistry, Molecular Biology and Biotechnology, University of Colombo, Colombo, LKA; 5 Department of Pharmacy and Pharmaceutical Science, University of Sri Jayawardhenapura, Colombo, LKA

**Keywords:** histological subtype, malignant dysphagia, palliative oesophageal stenting, post-stent survival, tumour location

## Abstract

Purpose

This study described post-stent survival and complication profiles in patients undergoing palliative oesophageal stenting for malignant dysphagia, to inform patient counselling.

Methods

An institutional audit reviewed palliative oesophageal stent insertions at Cambridge University Hospital (Apr 2020-Apr 2024). Patient demographics, complications, and survival were analyzed using SPSS (IBM Corp., Armonk, NY, USA) and Kaplan-Meier methods.

Results

Among 110 patients, 60.9% male (n=67); mean age 74.1 years, 95% CI 72.1-76.1), 93.6% (n=103) were deceased. Adenocarcinoma (n=68; 61.8%) and squamous cell carcinoma (n=32; 29.1%) were the most common histological subtypes, with tumours predominantly located in the lower and middle oesophagus. Thirty-six percent (n=39) received both chemotherapy and radiotherapy. Complications were stent migration (n=13;11.8%) and stent blockage (n=10;9.1%). Median post-stent survival was 162 days for squamous cell carcinoma, 97 days for adenocarcinoma, and 74 days for other types (log-rank p = 0.008); but did not differ significantly by tumour location (log-rank p = 0.73). In multivariable analysis, increasing age was associated with shorter post-stent survival (HR 1.05 per year, p = 0.001), while squamous cell carcinoma was associated with longer post-stent survival (HR 0.64, p = 0.037). Other variables were not independently associated with survival.

Conclusion

Palliative oesophageal stenting alleviates malignant dysphagia symptoms and has a known complication profile. Post-stent survival is linked to patient and tumour traits, underscoring its supportive role in prognostic counselling and post-procedure management.

Implications for cancer survivors

The findings highlight the role of oesophageal stenting in symptom relief rather than disease modification and may assist patients and clinicians in setting realistic expectations regarding prognosis and post-procedure care.

## Introduction

Oesophageal cancer is the sixth leading cause of cancer-related deaths worldwide [1,2]. Between 2015 and 2018, data from the UK indicated an annual incidence of over 9000 new cases of oesophageal cancer and approximately 8000 deaths attributed to this disease [3]. The majority of patients with oesophageal cancer present with advanced, incurable disease. Dubecz et al. (2012) described that among those with advanced disease, the median survival typically ranges from three to six months, with most patients having trouble swallowing (dysphagia) requiring intervention [4]. Although the optimal approach to palliating dysphagia has not been definitively established, available options include chemical and thermal ablation, self-expanding metal stents (SEMS), and radiotherapy (RT) or chemotherapy, either individually or in combination. Dai et al. (2014) suggest that in cases of advanced, incurable oesophageal cancer, SEMS insertion is an effective intervention for rapidly alleviating dysphagia [5]. Patients experience dysphagia before undergoing surgery, especially when large tumors obstruct the esophagus, resulting in notable weight loss and malnutrition [6,7]. The first attempt to treat oesophageal strictures using stents dates back to the 19th century, when Symonds introduced the first stent made of ivory and silver [8]. Stent therapy is often used in patients with a short life expectancy because of its ability to quickly improve malignant dysphagia [9]. However, stenting may not provide as effective pain relief as RT and carries a higher risk of adverse events [10]. Despite these considerations, stenting remains important for patients with a short life expectancy who strongly desire oral intake because it can rapidly alleviate dysphagia. To mitigate adverse events, particularly in patients with a history of RT, the use of low-radial-force stents should be considered. SEMs have been used for the palliative treatment of oesophageal carcinoma since 1990 [11]. One study done in 2012 stated that squamous cell carcinoma had shorter survival than adenocarcinoma and patients receiving adjuvant chemotherapy or chemoradiotherapy showed improved survival, likely due to treatment effect or patient selection [12]. Although post-stent survival in advanced oesophageal cancer is limited, stent placement can substantially improve quality of life by relieving dysphagia and allowing oral intake during the remaining disease course. Complications related to the stenting are worth evaluating to reduce them as well. This study aimed to describe post-stent survival, complication profiles, and associated prognostic factors in patients undergoing palliative oesophageal stenting. The study was designed as a retrospective, descriptive, and prognostic analysis rather than an interventional evaluation.

## Materials and methods

We aimed to evaluate the outcomes of palliative oesophageal stent insertion, focusing on patient characteristics, procedural complications, and survival. Patients who underwent oesophageal stent insertion between April 2020 and April 2024 at Cambridge University Hospital were identified from the local endoscopy database. All patients with oesophageal cancer referred for oesophageal stenting during the study period were included. Patients with unsuccessful stenting attempts, defined as cases where stent placement could not be completed due to technical, anatomical, or clinical reasons, were excluded from the analysis to focus on post-procedure outcomes.

All patients underwent oesophageal stent insertion with palliative intent following multidisciplinary team discussion. Indications included advanced incurable disease, poor performance status, or symptom-driven palliation. No patients were treated with radical intent at the time of stent placement.

Procedural techniques and stent selection were based on institutional practice; however, all procedures were performed by experienced endoscopists following standard clinical protocols.

The primary outcome, overall survival, was defined as the time (in days) from oesophageal stent insertion to death from any cause or last known follow-up, with patients alive at last follow-up censored.

The secondary outcomes included both immediate and delayed complications directly related to oesophageal stenting, such as perforation, stent migration, and obstruction.

Data on patient characteristics, including demographics, comorbidities, lesion site, and pathological diagnosis, were retrieved from electronic healthcare records. Stents were classified based on coverage (fully covered versus partially covered), irrespective of brand. Cases where stent brand was recorded without explicit documentation of coverage were treated as missing data, and procedural details were retrieved from electronic healthcare records. Post-procedure survival and complications were recorded and analyzed.

No pre-specified sample size was calculated, as all eligible patients within the study period were included. Descriptive statistics were expressed as percentages with 95% confidence intervals. Survival data were analyzed using the Kaplan-Meier method, and censored data were accounted for to provide a comprehensive estimate of survival duration. Survival outcomes were compared with log-rank tests. Statistical analyses were conducted using SPSS version 20 (IBM Corp., Armonk, NY, USA).

The study was conducted as part of a registered audit at Cambridge University Hospital in 2023 (approval number ID5891). Written informed consent was obtained from all patients prior to stent placement, including explicit consent for their data to be used in this analysis. This audit adhered to the ethical principles outlined in the Declaration of Helsinki, and measures were implemented to ensure data confidentiality and security.

## Results

We identified 110 patients who underwent palliative oesophageal stenting during the study period. Baseline sociodemographic characteristics, including age and gender distribution, are summarised in Table 1. The cohort was predominantly male (n=67;60.9%), with a mean age of 74.1 years (95% CI: 72.1-76.1). At last follow-up, 93.6% (n=103) of patients were deceased. Adenocarcinoma was the most common histological subtype (n=68; 61.8%), followed by squamous cell carcinoma (n=32; 29.1%). Tumours were most frequently located in the lower and middle oesophagus. Over one-third of patients received combined chemotherapy and radiotherapy, while 28.5% (n=33) did not receive any oncological treatment. Partially covered self-expanding metal stents were the most commonly used stent type. Baseline clinical and treatment characteristics are summarised in Table 1.

**Table 1:** Baseline clinical and treatment characteristics of patients undergoing palliative oesophageal stenting (n = 110) *Other histological types include metastatic, undifferentiated, neuroendocrine, and poorly differentiated tumours. CI: Confidence interval; SEMS: Self-expanding metal stent

Variable	Frequency	Percentage (%)
Gender		
Male	67	60.9
Female	43	39.1
Age (years)		
Mean age (95% CI)	74.1	(72.1 – 76.1)
Vital status at last follow-up		
Deceased	103	93.6
Alive	7	6.4
Histological subtype		
Adenocarcinoma	68	61.8
Squamous cell carcinoma	32	29.1
Other histological types*	10	9.1
Tumour location		
Lower oesophagus	41	37.3
Middle oesophagus	37	33.6
Gastro-oesophageal junction	25	22.7
Upper oesophagus	7	6.4
Oncological treatment received		
Chemotherapy and radiotherapy	39	36.4
Chemotherapy alone	27	25.2
Radiotherapy alone	11	10.3
No oncological treatment	33	28.5
Stent coverage type		
Partially covered SEMS	58	52.7
Fully covered SEMS	31	28.2
Coverage not specified	21	19.1

Immediate and delayed clinical outcomes following palliative oesophageal stent placement are summarised in Table 2. Delayed stent-related complications were more frequent than immediate post-procedural events, with stent migration (n=13; 11.8%) and stent blockage (n=10; 9.1%) being the most commonly observed adverse outcomes. Early post-procedural symptoms, including chest pain, abdominal pain, nausea, and aspiration-related events, occurred less frequently and were generally self-limiting. These findings demonstrate the spectrum and frequency of early and delayed complications following palliative oesophageal stenting and underscore the importance of anticipatory monitoring and post-procedure care in this patient population.

**Table 2:** Immediate and delayed clinical outcomes following palliative oesophageal stent placement. † Clinical outcomes were identified from endoscopy reports and electronic health records. Data on re-intervention (e.g. stent repositioning or replacement) were not consistently documented in a structured manner and therefore could not be analysed as a separate outcome.

Clinical Outcome^†^	Number of Patients	Percentage (%)
Immediate / Early Outcomes		
Chest pain	5	4.5
Abdominal pain	8	7.3
Nausea / vomiting	5	4.5
Aspiration	2	1.8
Aspiration pneumonia	3	2.7
Gastrointestinal bleeding	2	1.8
Cough	2	1.8
Delayed Outcomes		
Stent migration	13	11.8
Stent blockage / obstruction	10	9.1
Persistent or recurrent dysphagia	1	0.9
Vital Status at Last Follow-up		
Alive	7	6.4
Deceased	103	93.6

**Figure 1:**
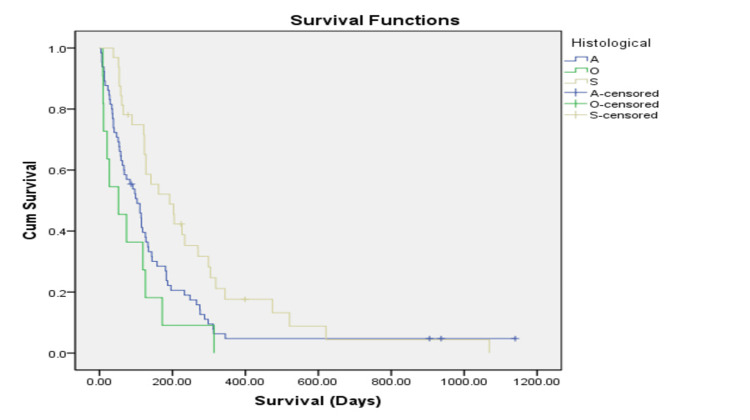
Comparing the survival of oesophageal cancer patients according to the histological subtype after palliative stent implantation using Kaplan-Meier (days) Cum Survival: Cumulative survival, A: Adenocarcinoma, O: Other types, S: Squamous cell carcinoma Log-rank χ² (2) = 9.6, p = 0.008, r = 0.30.

**Figure 2:**
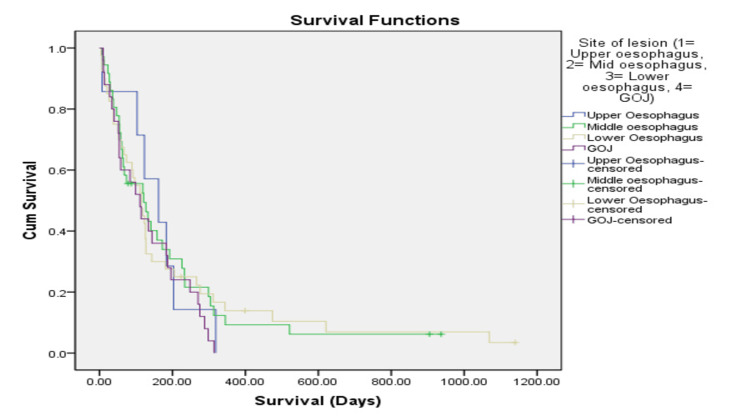
Comparison of post-stent survival according to site of lesion using Kaplan-Meier analysis. GOJ: gastro-oesophageal junction Log-rank χ²(3) = 1.3, p = 0.73, r = 0.11

Figures 1, 2 illustrate post-stent survival stratified by histological subtype and tumour location. Patients with squamous cell carcinoma demonstrated a longer median post-stent survival compared with those with adenocarcinoma and other histological subtypes (log-rank p = 0.008). Survival differences across tumour locations were not statistically significant (log-rank p = 0.73). These findings reflect underlying disease characteristics rather than a treatment effect of oesophageal stent placement. 

Descriptive post-stent survival varied across histological subtypes, tumour locations, and treatment groups (Table 3). Post-stent survival differed significantly according to histological subtype, log-rank χ²(2) = 9.6, p = 0.008, r = 0.30, but did not differ significantly according to site of lesion, log-rank χ²(3) = 1.3, p = 0.73, r = 0.11. Patients with squamous cell carcinoma demonstrated longer median post-stent survival compared with those with adenocarcinoma and other histological subtypes. However, differences in survival by tumour location and treatment modality were not statistically significant. These results are presented descriptively to provide prognostic context and should not be interpreted as evidence of treatment efficacy.

**Table 3:** Descriptive post-stent survival according to histological subtype, tumour location, and treatment modality. *Log rank test. ** Undifferentiated, poorly differentiated, metastasis, neuro-endocrine

	Mean (days)	Median (days)	95% confidence interval (days)	Significance
Histological type				Log-rank χ²(2) = 9.6, p = 0.008, r = 0.30*
Adenocarcinoma (n=68)	113	97	89-137	
Other types** (n=10)	96	74	46-146	
Squamous cell carcinoma (n=32)	230	162	148-312	
Site of lesion				Log-rank χ²(3) = 1.3, p = 0.73, r = 0.11
Upper oesophagus (n=7)	157	162	85-229	
Middle oesophagus (n=37)	138	119	96-180	
Lower oesophagus (n=41)	155	113	89-221	
Gastro-oesophageal junction (n=25)	132	111	92- 172	
Type of treatment				
None (n=33)	130	59	80-180	
Radiotherapy (n=50)	133	123	91-175	
Chemotherapy (n=66)	88	87	68-108	

Table 4 presents the results of the multivariable Cox proportional hazards regression analysis evaluating factors associated with survival following palliative oesophageal stenting. After adjustment for potential confounders, age was significantly associated with increased mortality, with each additional year conferring a higher risk of death (HR = 1.05, 95% CI: 1.02-1.08, p = 0.001). Histological subtype remained an independent predictor of survival; patients with squamous cell carcinoma demonstrated significantly better survival compared to those with adenocarcinoma (HR = 0.64, 95% CI: 0.42-0.97, p = 0.037). Gender, stent location, and treatment modality did not significantly impact post-stent survival in the adjusted model. Similarly, the occurrence of major complications, such as stent migration, was not independently associated with poorer outcomes (HR = 1.31, 95% CI: 0.75-2.27, p = 0.33). These findings suggest that, among patients undergoing palliative oesophageal stenting, histological subtype and increasing age are key independent predictors of survival, while other clinical and procedural variables appear less influential after adjustment. Multivariable analysis was performed to identify prognostic factors associated with post-stent survival rather than to assess the effect of stent placement on survival.

**Table 4:** Multivariable Cox proportional hazards regression analysis of factors associated with post-stent survival in patients undergoing palliative oesophageal stenting. RT: Radiotherapy, ref: reference to

Variable	Beta	Exp (Beta) (HR)	95% CI for Exp (Beta)	P-value
Age (per year increase)	0.05	1.05	1.02 – 1.08	0.001
Male (vs. female)	0.20	1.22	0.85 – 1.76	0.28
Squamous cell carcinoma (ref: adenocarcinoma)	-0.45	0.64	0.42 – 0.97	0.037
Lower esophagus (ref: upper)	0.17	1.19	0.70 – 2.04	0.52
Both chemo + RT (ref: none)	-0.38	0.68	0.44 – 1.07	0.097
Chemotherapy only (ref: none)	0.02	1.02	0.67 – 1.54	0.94
Radiotherapy only (ref: none)	-0.10	0.91	0.49 – 1.70	0.78
Stent migration (yes vs. no)	0.27	1.31	0.75 – 2.27	0.33

## Discussion

This study describes post-stent survival and complication profiles in patients undergoing palliative oesophageal stenting for oesophageal malignancies. It also highlights the complications that commonly occur, which are comparable with previous studies on oesophageal stent insertions. These factors should be considered during shared decision-making and patient counselling prior to oesophageal stent insertion as a palliative intervention. As expected, the cohort was predominantly male with a mean age of 74 years [13]. Our study showed that 94% (n=103) of the patients were deceased at the time of assessment. This suggests a relatively low survival rate, which is consistent with the generally poor prognosis associated with oesophageal cancer, especially in advanced stages. Adenocarcinoma (n=68; 59%) and squamous cell carcinoma (n=32; 29%) are identified as the most common types of oesophageal cancer in this patient cohort. This distribution aligns with global trends, where adenocarcinoma is more prevalent in Western countries, often associated with gastroesophageal reflux disease (GERD) [14]. The lower esophagus (n=41; 37%) is noted as the most frequent site for cancerous lesions. This finding is consistent with epidemiological studies showing a higher incidence of adenocarcinoma in the lower esophagus, potentially related to GERD and Barrett's [15]. Self-expandable metal stents (partially covered) were the most commonly employed type for managing oesophageal obstruction (34%).

Complications after stenting

The reported incidence of stent migration (n=13; 12%) aligns with findings from previous studies. For example, a study by Siersema et al. (2008) reported stent migration rates ranging from 10% to 50% [16]. Other studies have also highlighted stent migration as a significant complication, emphasizing the importance of proper stent selection, sizing, and deployment techniques to minimize this risk. The prevalence of stent blockage (n=10; 9%) is consistent with data from clinical trials and systematic reviews. Knyrim et al. (1993) reported rates of stent obstruction ranging from 10% to 30%, primarily attributed to tumor ingrowth or overgrowth [17]. The incidence of chest pain, nausea/vomiting, aspiration, dysphagia, gastrointestinal bleeding, aspiration pneumonia, and cough observed in the study aligns with broader literature on stent-related complications. Conio et al. (2018) documented abdominal pain rates ranging from 10% to 20% in patients receiving oesophageal stents for palliation of malignant strictures [18]. Knyrim et al. (1993) observed chest pain in 12% of patients undergoing stent placement for malignant oesophageal obstruction [17]. Nausea and vomiting can occur due to stent-related factors and gastric outlet obstruction or altered gastric motility. Studies by Jong Ho Moon et al. (2014) and Dougherty et al. (2017) have documented comparable rates of nausea and vomiting following oesophageal stent placement for malignant strictures [19,20]. Research by Hirdes et al. (2013) and Siersema (2008) has reported similar rates of aspiration and aspiration pneumonia following oesophageal stenting [21,16]. Dysphagia, or difficulty swallowing, may persist or worsen post-stent placement due to stent-related issues or disease progression. Siersema (2008) documented dysphagia rates ranging from 1% to 20% in patients receiving oesophageal stents for palliation of malignant strictures [16]. Gastrointestinal bleeding can occur secondary to stent-related trauma or tumor erosion. Studies by Homs et al. (2004) and Bethge et al. (2008) have reported similar rates of gastrointestinal bleeding following oesophageal stent placement [22,23]. Cough is a recognized symptom following oesophageal stent placement, often attributed to airway irritation or unresolved dysphagia. Bergquist et al. (2005) and Kim et al. (2010) have documented cough as a common post-stent symptom in patients with malignant oesophageal strictures [24,25].

Adenocarcinoma of the esophagus is commonly associated with poorer survival outcomes compared to other histological types. Several studies support this observation. For example, a study by Homs et al. (2004) examined the outcomes of stent placement in patients with malignant oesophageal obstruction. They found that patients with adenocarcinoma had a median survival of approximately three months, which aligns with the presented data [22]. The longer post-stent survival observed in patients with squamous cell carcinoma is consistent with findings from other studies and likely reflects differences in tumour biology and disease behaviour rather than the effect of stent placement. It can be due to more involvement of squamous cancer in the upper lesions [26]. Squamous cell carcinoma is generally associated with a more favorable prognosis compared to adenocarcinoma and other subtypes, likely due to differences in tumor biology and responsiveness to treatment [27].

The association between lesion site and post-stent survival in patients undergoing oesophageal stent placement is an important consideration in the clinical assessment of oesophageal cancer. The observed differences in survival durations across anatomical segments, particularly lower lesions at the gastro-oesophageal junction and lower oesophagus showing shorter median survival compared to upper and middle oesophageal lesions, align with existing literature on this topic. Several studies have investigated the association between lesion site and prognosis in oesophageal cancer. For example, Lagergren and colleagues (2017) noted that tumour location influences disease progression and treatment response, with lower oesophageal and gastro-oesophageal junction lesions often posing greater challenges in terms of therapeutic efficacy and patient outcomes [6]. Siersema (2008) explored treatment options for oesophageal strictures, acknowledging the distinct challenges associated with lesions located at different anatomical sites and their implications for stent placement and palliative care [16].

Importantly, in our multivariable Cox proportional hazards regression analysis, both increasing age and histological subtype remained independent predictors of survival following palliative stent placement. Older patients had a significantly higher risk of mortality, while those with squamous cell carcinoma demonstrated improved survival compared to adenocarcinoma, independent of other clinical factors. Other variables, including gender, tumor location, treatment modality, and occurrence of stent-related complications, were not found to be significant predictors of survival in the adjusted model. These findings reinforce that histological subtype and patient age play a central role in prognosis, regardless of treatment or technical aspects of the procedure. These associations should be interpreted as prognostic indicators reflecting patient and tumour characteristics rather than as evidence of a survival benefit attributable to stent placement.

Based on these findings, oesophageal stent insertion should be regarded as a supportive intervention for dysphagia associated with malignancy rather than a disease-modifying treatment, because it helps to maintain nutrition, allowing a patient to enjoy the taste of food and prevent tube feeding. It does not alter the activity of the tumour or the progression of it. This study will help to answer the questions which patient would ask before accepting a stent or requesting a stent.

Strengths

This study has several strengths. It includes a relatively large real-world cohort of patients undergoing palliative oesophageal stenting over a defined time period. The use of survival analysis and multivariable Cox regression provides clinically relevant prognostic insights. Additionally, the inclusion of both complication profiles and survival outcomes offers a comprehensive evaluation of post-stent clinical trajectories.

Limitations

This study did not account for depth of tumour invasion, presence of distant metastases, or lymph node involvement when comparing post-stent survival across histological subtypes. The retrospective design may have introduced selection and information biases, as data were obtained from existing medical records rather than collected prospectively. In addition, this was a single-centre study, which may limit the generalisability of the findings to other populations and clinical settings. The relatively small sample size may have reduced statistical power and limited detailed subgroup analyses.

Although post-stent survival was analysed, important prognostic factors such as performance status and comorbidities, which could substantially influence survival, were not consistently available for evaluation. Variations in stent types and procedural techniques over time may also have affected complication rates and contributed to variability in outcomes. Furthermore, residual confounding due to unmeasured variables cannot be excluded. Finally, variability in follow-up duration and incomplete documentation of certain clinical parameters may have influenced the observed results.

## Conclusions

Palliative oesophageal stenting is a supportive intervention for the management of malignant dysphagia, with a predictable profile of early and delayed complications, most commonly stent migration and blockage. Post-stent survival in this cohort reflected underlying patient and tumour characteristics rather than a treatment effect of stent placement. In multivariable analysis, increasing age and histological subtype, particularly squamous cell carcinoma, were associated with differences in post-stent survival, while gender, tumour location, oncological treatment modality, and stent-related complications were not independently associated. These findings may assist clinicians in prognostic counselling, informed consent, and anticipatory management of complications in patients undergoing palliative oesophageal stenting.
